# A comparative study of robotics and laparoscopic in minimally invasive pancreatoduodenectomy: A single-center experience

**DOI:** 10.3389/fonc.2022.960241

**Published:** 2022-10-05

**Authors:** Ke Zong, Kai Luo, Kunlun Chen, Jianwen Ye, Wentao Liu, Wenlong Zhai

**Affiliations:** Departments of Hepatobiliary Surgery, Zhengzhou, The First Affiliated Hospital of Zhengzhou University, Zhengzhou, China

**Keywords:** minimally invasive surgery, pancreatoduodenectomy, robotic surgery, laparoscopic surgery, surgical complication

## Abstract

**Objective:**

To retrospectively compare the short-term benefits of robotic surgery and laparoscopic in the perioperative period of minimally invasive pancreatoduodenectomy (MIPD).

**Methods:**

This retrospective analysis evaluated patients who underwent laparoscopic pancreatoduodenectomy (LPD) or robotic pancreatoduodenectomy (RPD) from March 2018 to January 2022 in the First Affiliated Hospital of Zhengzhou University (Zhengzhou, China). Perioperative data, including operating time, complications, morbidity and mortality, estimated blood loss (EBL), and postoperative length of stay, were analysed.

**Result:**

A total of 190 cases of MIPD were included, of which 114 were LPD and 76 were RPD. There was no significant difference between the two groups in gender, age, previous history of upper abdominal operation, jaundice (>150 µmol/L), or diabetes (*P* > 0.05). The conversion rate to laparotomy was similar in the LPD and RPD groups (5.3% vs. 6.6%, *P* = 0.969). A total of 179 cases of minimally invasive pancreatoduodenectomy were successfully performed, including 108 cases of LPD and 71 cases of RPD. There were significant differences between the laparoscopic and robotic groups in operation time [mean, 5.97 h vs. 5.42 h, *P* < 0.05] and postoperative length of stay [mean, 15.3 vs. 14.6 day, *P* < 0.05]. No significant difference was observed between the two groups in terms of EBL, intraoperative transfusion, complication rate, mortality rate, or reoperation rate (*P* > 0.05). There were no significant differences in pathological type, number of lymph nodes harvested, or positive lymph node rate (*P* > 0.05).

**Conclusion:**

RPD had an advantage compared to LPD in reduced operation time and postoperative length of stay, technical feasibility, and safety.

## Introduction

In the near century since Whipple first reported pancreatoduodenectomy in 1938 (1), pancreatoduodenectomy has become a standard procedure for periampullary tumours. With the development of laparoscopic technology and internal closure devices and the wide application of energy instruments, minimally invasive pancreatectomy has gradually spread around the world. However, due to the particularity of pancreatic anatomy, the development of minimally invasive pancreatic surgery has not been as smooth as that of urologic, obstetric, gynaecologic, and gastrointestinal surgeries. Gagner first reported laparoscopic pancreatoduodenectomy (LPD) in 1994 (2), and many surgeons and centers have performed LPD. At present, due to the technical requirements and limitations of LPD, minimally invasive distal pancreatectomy (MIDP) has been more widely performed by pancreatic surgeons. Thus, MIDP provides valuable experience in minimally invasive pancreatectomy for the development of MIPD. Laparoscopic total pancreatectomy (LDP) and robotic total pancreatectomy (RDP) can reduce the length of the postoperative hospital stay, and the complication rate and mortality are also acceptable (3–5).

However, in some high-flow hospitals, the complication rate and mortality of LPD can reach the same level as those of open pancreatoduodenectomy (OPD), and LPD can even have some advantages in reducing estimated blood loss and length of postoperative hospital stay (6–9). However, due to the limitations of the equipment, laparoscopic instruments have limited mobility in the cavity, which affects the operation, especially in the digestive tract reconstruction stage. Thanks to the invention of robotic surgery systems, Italian surgeon Giulianotti took the lead in applying robotic surgery to robotic pancreatoduodenectomy (RPD) in 2003 (10). The robotic system has a more flexible arm, a clearer surgical field of view, and three-dimensional visualization and aids in the elimination of tremors. Therefore, the surgeon can control the instrument more finely and flexibly (11). Compared with laparotomy, RPD has achieved similar results to LPD, such as more precise operation during surgery, no difference in perioperative complications, and shorter hospital stays (12–15). Although minimally invasive pancreatoduodenectomy has developed rapidly, there are few comparative studies on laparoscopic and robotic pancreatoduodenectomy and fewer experiences in a single center, mainly because hospitals that have conducted both methods are rare. As a hospital performing both LPD and RPD, we aimed to analyse the advantages and disadvantages of laparoscopic and robotic pancreatoduodenectomy in the perioperative period through a retrospective study.

## Materials and methods

### General data

The following inclusion criteria were used: (1) MIPD for periampullary benign or malignant tumours; (2) no distant metastasis; (3) no invasion of the common hepatic or superior mesenteric arteries, other organs in the abdominal cavity, or the abdominal aorta, inferior vena cava, and other large vessels; and (4) adequate general medical conditions for general anaesthesia with pneumoperitoneum. The following exclusion criteria were used: (1) inability to tolerate long-term pneumoperitoneum and anaesthesia; (2) distant metastasis; (3) invasion of the artery and other abdominal organs; (4) operations performed by other doctors; (5) neoadjuvant therapy; and (6) tumour size too large to conduct MIPD according to the surgeons’ experience. After doctors informed the patients of the advantages and disadvantages of LPD and RPD, the surgical procedure was selected according to the wishes of the patients. All patients signed informed consent forms before the operation. The chief surgeon performing robotic surgery was Professor Wenlong Zhai, and the assistant was either Ke Zong, Jianwen Ye, or Wentao Liu. Laparoscopic surgery was performed by Professor Wenlong Zhai or Professor Kunlun Chen, and the assistant was one of the above doctors chosen at random. The two surgical methods were started during almost the same period. We reviewed preoperative data, such as gender, age, previous history of upper abdominal operation, jaundice (last laboratory results before operation, total bilirubin >150 µmol/l), and diabetes. We reviewed all eligible case data from March 2018, when the hospital started performing minimally invasive pancreatoduodenectomies, to January 2022. A total of 190 MIPD cases were included. There were 113 men and 77 women, with a mean age of 58.1 ± 1.5 (21-82) years. There were 114 cases in the laparoscopic group and 76 cases in the robotic group. This study was conducted in accordance with the Declaration of Helsinki (revised in 2013) and approved by the Institutional Review Board.

### Method of operation

#### Robotic pancreatoduodenectomy

**Figure 1:**
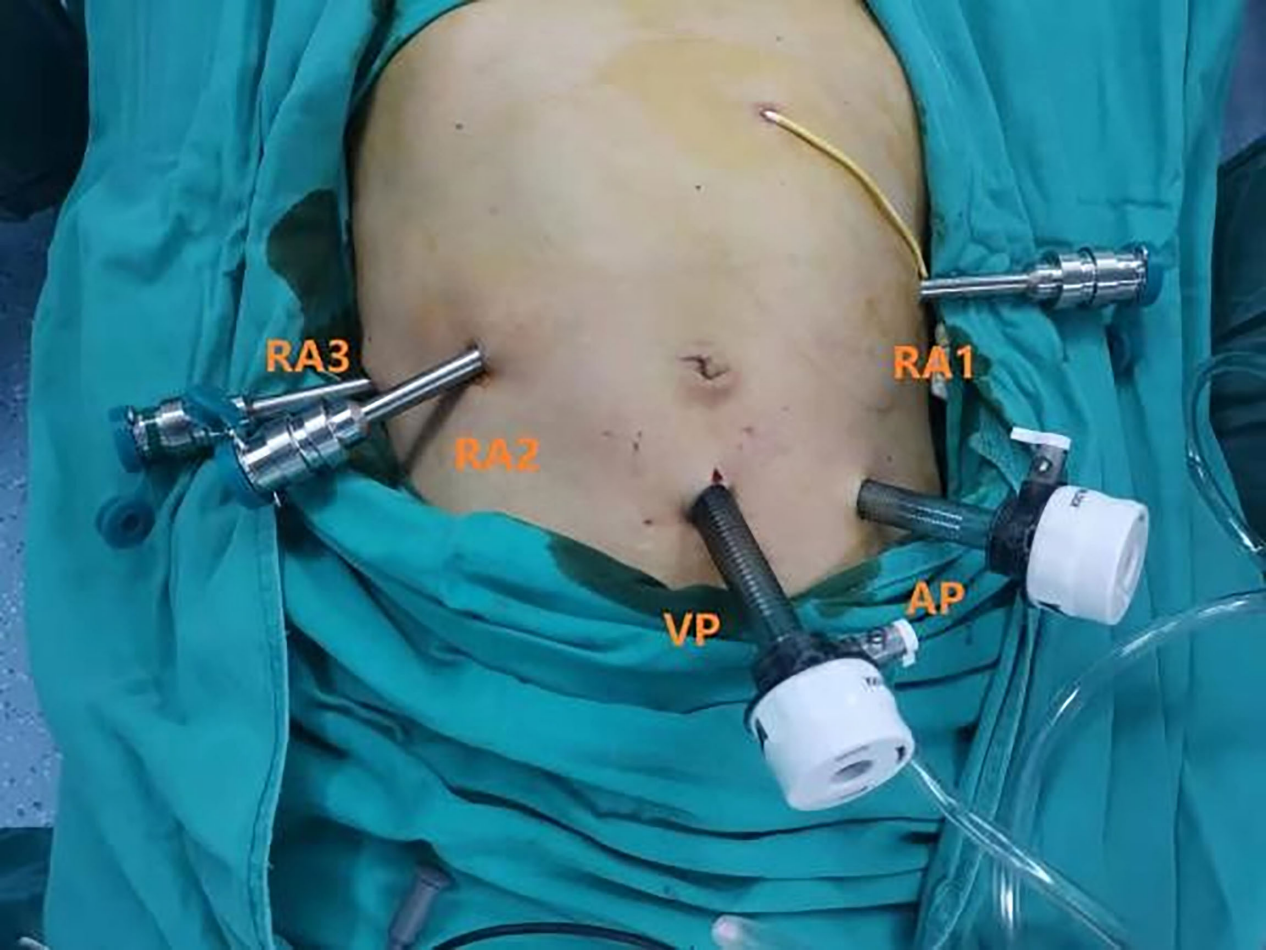
Robotic ports placement.

The patient was placed in a supine 30° reverse Trendelenburg position with their legs apart and a slight right-side tilt (approximately 10°). The surgeons performed all robot-assisted surgeries with the Da Vinci Si Surgical System (Intuitive Surgical, Sunnyvale, CA, USA). Following standard procedure, we used a pneumoperitoneum needle to establish pneumoperitoneum at 12–15 mmHg under the left costal arch, which is a safer site to avoid secondary damage. Then, a 12-mm trocar (view port) was successfully placed in the subumbilical (2–3 mm) site for pneumoperitoneum imaging and laparoscopy. The needle was removed, and the pneumoperitoneum tube was attached to the view port. After laparoscopic exploration, RA3 (robot arm 3), RA2 (robot arm 2), and RA1 (robot arm 1) trocars were placed in the right axillary midline, clavicular midline, and left clavicular midline, respectively. A 12-mm assistant trocar was placed between the view port and the RA1 trocar. A camera was placed in the view port. The assistant 12-mm trocar was used by the assistant surgeon to pass the needles and manage the suction irrigator and endostapler **(** **Figure 1**).

**Figure 2:**
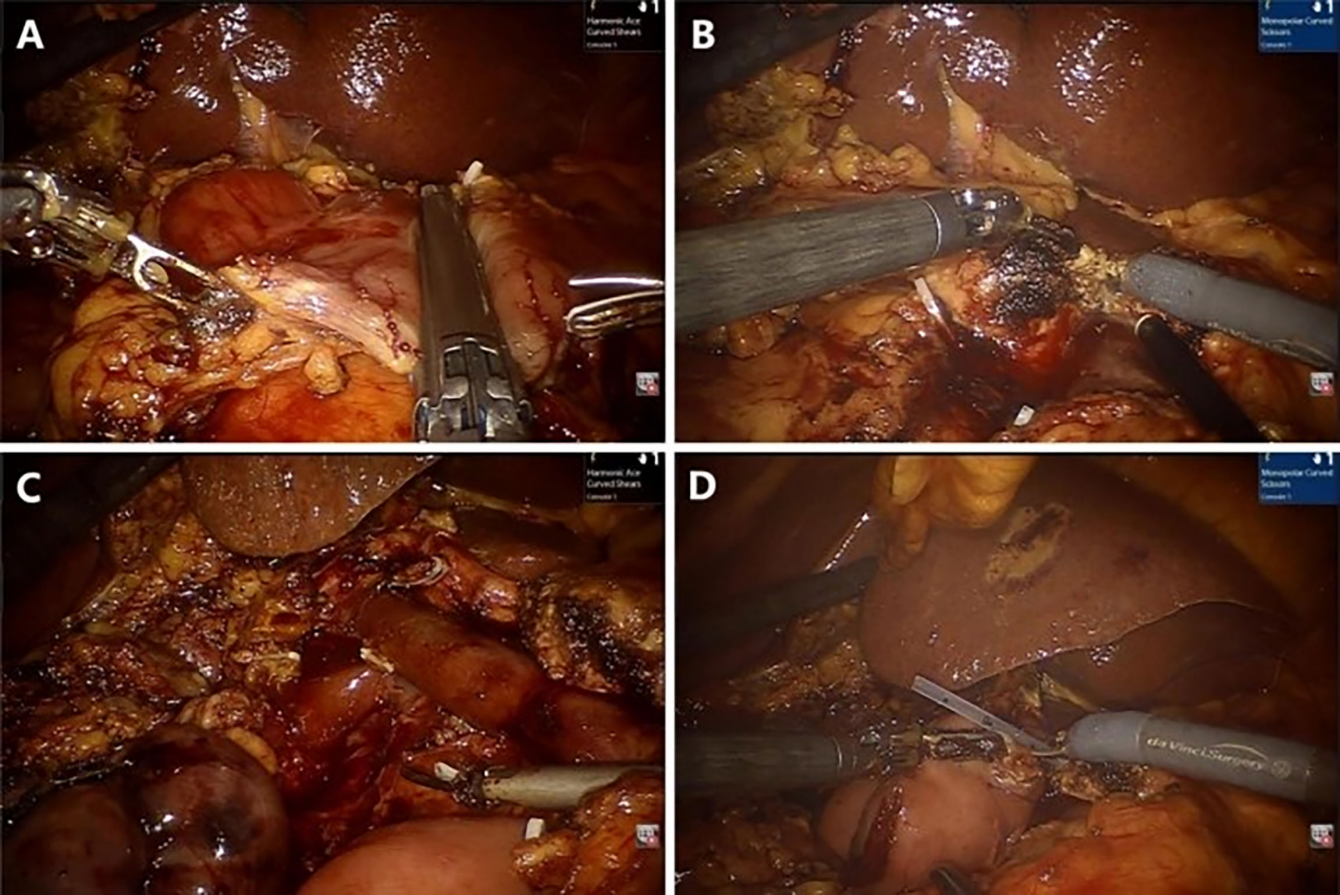
Representative photographs of the RPD: **(A)** Transection of the stomach. **(B)** Transection of the pancreas. **(C)** Complete resection. **(D)** Pancreaticojejunostomy anastomosis.

The gastrocolic ligament was opened for preliminary exploration of the pancreas, usually with electric scissors. The superior mesenteric vein (SMV) was found along the inferior margin of the pancreas, and the Henle’s trunk was ligated and cut off. The surgeon performed an extended Kocher manoeuvre to mobilize the transverse duodenum from the ligament of Treitz beneath the SMV. The common hepatic artery lymph nodes were dissected, and the right gastric artery and gastroduodenal artery were ligated at an appropriate length. Then, the small intestine, stomach, pancreas, and bile duct were cut off **(** **Figure 2A, B**). Complete resection of the uncinate process of the pancreas with electric scissors was performed **(** **Figure 2C**). The distant small intestine was moved to the right region through the original duodenal aperture to reconstruct the digestive tract by pancreatojejunostomy anastomosis **(** **Figure 2D**), hepaticojejunostomy anastomosis, and gastrojejunostomy anastomosis in order. A 5-cm curved periumbilical incision was made to remove the specimen, and the robotic system was removed.

#### Laparoscopic pancreatoduodenectomy

**Figure 3:**
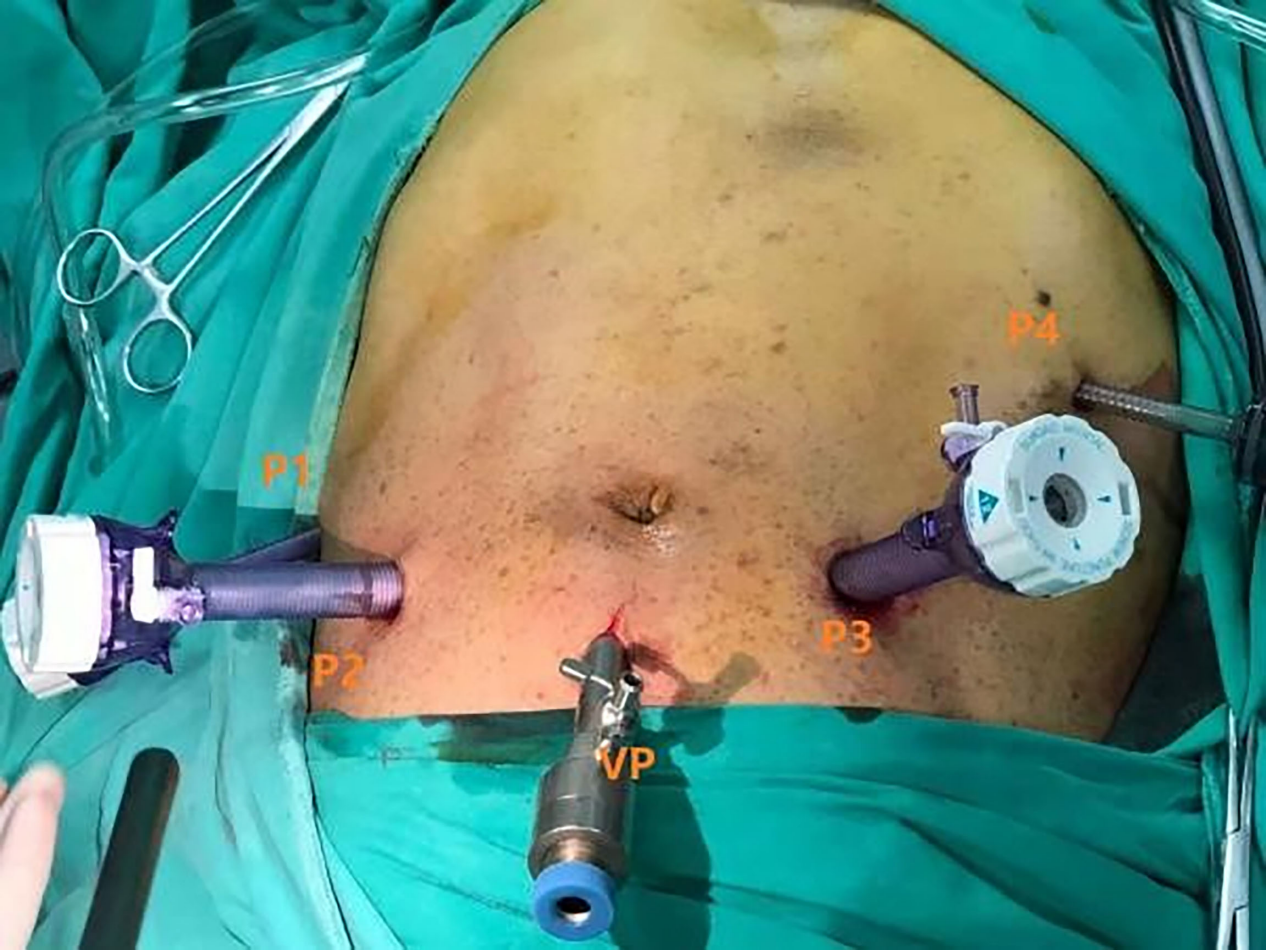
Laparoscopic port placement.

**Figure 4:**
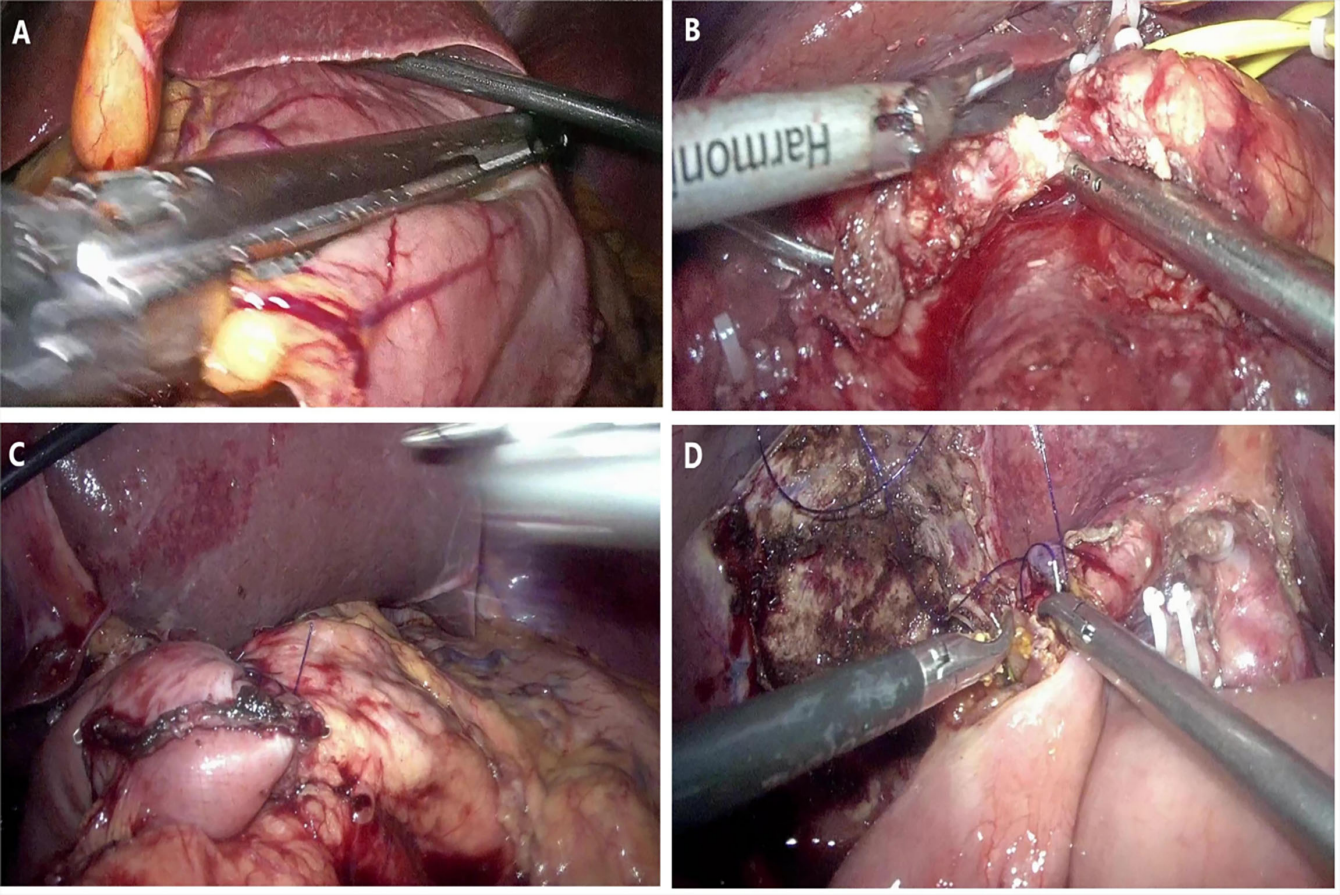
Representative photographs of the LPD: **(A)** Transection of stomach. **(B)** Transection of pancreas. **(C)** Pancreaticojejunostomy anastomosis. **(D)** Hepaticojejunostomy anastomosis.

The patient position was the same as that for RPD. A 12-mm trocar was placed in the subumbilical (1 cm) site for pneumoperitoneum imaging and laparoscopy. The scope was inserted to explore the abdominal cavity to exclude distant metastasis, and a V-shaped trocar arrangement was placed (**Figure 3**). LPD was performed with an ultrasonic scalpel, and the remaining operations were the same as those for RPD **(** **Figure 4** **)**.

### Perioperative observation and complication standard

The number of conversions was counted in both groups. The intraoperative and postoperative data of the patients who were not converted to laparotomy were recorded, and the pathological data of all cases were reviewed. Postoperative pancreatic fistula (PF) (16), delayed gastroparesis (DGE) (17), and haemorrhage (PPH) (18) were defined according to the International Research Group on Pancreatic Surgery (ISGPS) criteria.

### Statistical methods

The statistical software SPSS 19.0 was used for statistical analysis. Normality tests and homogeneity tests of variance were carried out for the data indicators of the study, and mean ± SD was used to describe the normally distributed continuous variables. The differences between the two groups of normally distributed data were compared by independent sample *t* tests. Qualitative data were used to calculate the composition ratio and rate, and the χ^2^ test or Fisher’s exact probability test was used to compare the differences between groups. Differences at *P*<0.05 were considered statistically significant.

## Results

### Preoperative general data and number of conversions

A total of 190 patients underwent minimally invasive pancreatoduodenectomy, including 116 in the laparoscopy group and 76 in the robot group. There was no significant difference between the two groups in gender, age, number of patients with upper abdominal surgical history, number of patients with preoperative bilirubin greater than 150 µmol/l, or number of patients with diabetes (**Table 1**).

**Table 1:** General data and conversion of preoperation.

	LPD (n = 114)	RPD (n = 76)	*P*-value
Gender, M/F	77/37	36/40	0.513
Age, mean ± SD (range), years	58.1 ± 1.4 (21~82)	58.2 ± 1.7 (31~74)	0.916
Previous history of upper abdominal operation	5 (4.4%)	6 (8.5%)	0.591
Jaundice	17 (14.9%)	7 (9.2%)	0.205
Diabetes	20 (17.5%)	8 (13.8%)	0.865
Conversion	6 (5.3%)	5 (6.6%)	0.969

Jaundice: last laboratory results before operation total bilirubin >150 µmol/l. Results are presented as number (%), unless otherwise indicated.

There were six cases (5.3%) in the laparoscopic group and five cases (6.6%) in the robot group that were converted to laparotomy. There was no significant difference between them (*P* = 0.969) **(** **Table 1** **)**. Therefore, a total of 179 cases successfully completed minimally invasive surgery.

### Perioperative results without conversion

Among the 179 patients who successfully completed minimally invasive surgery, 108 underwent LPD and 71 underwent RPD. There was no significant difference in EBL or blood transfusion during the operation between the two groups. The operation time of RPD was significantly shorter than that of LPD (5.42 ± 0.12 vs. 5.97 ± 0.14 h, *P* = 0.023) **(** **Table 2** **)**. In terms of postoperative complications, there was no significant difference in the incidence of pancreatic fistula, bile leakage, delayed gastric emptying, gastrointestinal bleeding, or abdominal bleeding. There was one case of reoperation in the laparoscopic group and one case in the robotic group, with no significant difference; there were no deaths within 30 days in either group. The postoperative hospital stay in the LPD group (15.3 ± 0.8 days) was longer than that in the RPD group (14.6 ± 1.1 days), and the difference was statistically significant (*P* = 0.034) **(** **Table 2** **)**.

**Table 2:** Perioperative results without conversion.

	LPD (n = 108)	RPD (n = 71)	*P*-value
Operation time, mean ± SD (range), h	5.97 ± 0.14 (4.0~8.5)	5.42 ± 0.12 (3.3~7.0)	0.023
Estimate blood loss, mean ± SD (range), mL	378.5 ± 37.1 (100~1,600)	309.0 ± 32.7 (50~1,000)	0.265
Intraoperative blood transfusion	21 (18.5%)	9 (12.7%)	0.345
Pancreatic fistula			
Biochemical leak	23 (21.3%)	16 (22.5%)	0.680
B	15 (13.9%)	5 (7.0%)	0.220
C	2 (1.9%)	3 (4.2%)	1
Bile leakage	14 (13.0%)	4 (5.6%)	0.313
Delayed gastric empty	12 (11.1%)	8 (11.3%)	1
Gastrointestinal bleeding	7 (6.5%)	2 (2.8%)	0.255
Abdominal bleeding	2 (1.9%)	1 (1.4%)	1
Reoperation	1 (0.9%)	1 (1.4%)	0.398
Mortality within 30 days	1 (0.9%)	0 (0%)	1
Postoperative hospital stay, mean ± SD (range), days	15.3 ± 0.8 (8~48)	14.6 ± 1.1 (7~40)	0.034

Results are presented as number (%), unless otherwise indicated.

### Pathological results without conversion

In the robotic group, the proportion of pancreatic lesions was lower and bile duct lesions was higher, but the difference was not significant compared with LPD (*P* = 0.098). Moreover, subgroup analysis showed that in the three groups, there was no significant difference in the pathological diagnosis of the lesions. The results of lymph node harvest and lymph node positive rate of the two surgical methods were similar. Although the robotic group harvested more lymph nodes, the difference was not statistically significant **(** **Table 3** **)**.

**Table 3:** Pathological results without conversion.

	LPD (n = 108)	RPD (n = 71)	*P*-value
Pancreatic lesions	35 (32.4%)	19 (26.8%)	0.098
Duodenal and papillary lesions,	35 (32.4%)	23 (32.4%)	
Bile duct lesions	38 (35.2%)	29 (40.8%)	
Number of lymph nodes harvest, mean ± SD (range)	9.07 ± 0.6 (1~25)	9.97 ± 0.90 (3~24)	0.722
With positive lymph nodes	18 (16.7%)	11 (15.5%)	0.985
**Subgroup pathology**			
	LPD	RPD	*P*-value
Pancreatic lesions	n = 35	n = 19	
Malignant	22 (62.9%)	13 (68.4%)	1
Benign	10 (28.6%)	5 (26.3%)	
Pancreatitis	3 (8.6%)	1 (5.3%)	
Duodenal and papillary lesions,	n = 35	n = 23	
Malignant	31 (88.6%)	21 (91.3%)	1
Benign	3 (8.6%)	2 (8.7%)	
Duodenal papillitis	1 (2.9%)	0	
Bile duct lesions	n = 38	n = 29	
Malignant	34 (89.5%)	27 (93.1%)	1
Benign	3 (7.9%)	2 (6.9%)	
Cholangitis	1 (2.6%)	0	

Results are presented as number (%), unless otherwise indicated. Pancreatic lesions including benign and malignant tumours of the pancreas and chronic pancreatitis; bile duct lesions including benign and malignant tumours of the bile duct and cholangitis; duodenal and papillary lesions including benign duodenal malignancies, duodenal inflammation, malignant and benign duodenal papillary tumours, and papillitis.

## Discussion

With the improvement of laparoscopic technology, the accumulation of experience, and the progress of laparoscopic equipment, laparoscopic surgery has developed rapidly and become a routine surgical procedure in medical centers. However, compared with laparoscopic gastrointestinal and liver operations, LPD has not been widely performed. There may be several reasons for this: (1) the procedure of pancreatoduodenectomy is cumbersome; not only are many organs removed but also several digestive tract reconstructions are needed, the operation is complex, and the postoperative management is difficult; (2) the learning curve is long, and it is difficult for many surgeons to cross the technical gap of LPD; and (3) it is difficult to complete lymph node dissection, accurate dissociation, anastomotic reconstruction, and other operations under “chopstick-like operation” conditions and the two-dimensional laparoscopy field of view. To break through the limitations of LPD, Giulianotti et al. (10) reported robotic pancreatoduodenectomy for the first time in 2003. The improvement of the Da Vinci robotic surgery system is mainly reflected in the following three aspects: (1) the visual is a three-dimensional imaging and enlarged 10–15 times; (2) the robot arm is more flexible, and the tremor of the surgeon can be filtered; and (3) the hand–eye coordination of the operator accelerates the learning process. However, it also has some defects, such as a higher cost and a lack of direct force feedback.

At present, MIPD has been widely performed in many medical centers. Compared with traditional open surgery, LPD and RPD have certain advantages (6, 12–15, 19, 20). Croome et al. (6) included 108 cases of laparoscopy and 214 cases of laparotomy and showed that the laparoscopic group had complication rates similar to those of the laparotomy group, but the LPD group had a shorter postoperative hospital stay. In several studies comparing robotic surgery with open surgery, it was found that the blood transfusion rate during surgery and length of postoperative hospital stay were lower in the RPD group (12–14). The above articles also show that minimally invasive pancreatoduodenectomy and laparotomy can achieve similar results in terms of lymph node harvest and blood loss (6, 20). However, the comparison of perioperative results between RPD and LPD still needs further study. This study aims to evaluate the short-term benefits of the two surgical methods.

In this study, we conducted a retrospective comparison between LPD and RPD. The results showed that although robotic surgery had advantages in reducing intraoperative bleeding, the difference was not statistically significant compared with LPD (378.5 ± 37.1 vs. 309.0 ± 32.7 ml, P > 0.05), which was similar to the results of other studies (21–23). Compared with the LPD group, the operation time and length of hospitalization were significantly shorter in the RPD group (5.97 ± 0.14 vs. 5.42 ± 0.12 h, P < 0.05). Kim et al. (21) found that after propensity score matching analysis, the operation time and length of postoperative hospital stay of RPD were shorter than those of laparoscopy, with significant differences (411.6 vs. 452.6 min, P = 0.001; 14.6 vs. 11.9 days, P = 0.027). Similar results were obtained by Zhao et al. (24). The results of Park et al. (23) showed that the operation time of the RPD group was significantly shorter than that of the LPD group (400.40 vs. 352.15* min*, P = 0.003), but the length of hospital stay was basically the same. Compared with laparoscopic surgery, the robotic system needs to be assembled before the operation, but the operation time of RPD is still significantly shorter than that of LPD, which indicates that the more flexible mechanical arms make knotting and suturing easier and thus shorten the operation time. The research of Gall et al. (25) also shows that the accuracy and efficacy of the robotic suture and knot are higher and better than LPD. The three-dimensional refined field of view and tremor elimination are more conducive to accurate dissection, reduced secondary damage, and accelerated digestive tract reconstruction, which make robotic operations more advantageous in complex surgery (24). The above conclusions were also verified in the results analysis of two randomized controlled trials (RCTs) of pancreaticoenterostomy and biliary anastomoses based on biological tissue models; that is, compared with 2D and 3D laparoscopy, robotic surgery is more efficient in anastomosis (26).

Regarding complications, there was no significant difference in all-grade pancreatic fistula, bile leakage, delayed gastric emptying, gastrointestinal bleeding, or abdominal bleeding between the two groups (P > 0.05). Among the more serious complications, no significant difference was found in secondary operation or 30-day mortality (P > 0.05). This is the same as the results of many studies (21, 24, 27, 28). The literature written by Korean surgeons (22) showed that the incidence of complications was similar after comparative analysis between LPD and RPD. Subgroup analysis showed that in patients with a soft pancreas and normal pancreatic duct, the postoperative pancreatic fistula rate was also basically the same, which may be related to their completion of 207 LPD operations, and the procedure was well developed. Another study aimed to analyse the difference between OPD and MIPD in patients with pancreatic duct dilatation and showed that patients with pancreatic duct diameters ≥3 mm can obtain more benefits from MIPD, which is associated with a lower incidence of postoperative complications and shorter hospital stays (29).

Several multicenter studies have reported that robotic surgery has more advantages in reducing conversion to laparotomy, which indicates that the robot’s clear field of view and handheld operation are helpful to avoid conversion (15, 21, 30, 31). However, in our study, it was found that the difference between the two groups in conversion rate was very small and not statistically significant (six cases in LPD, 5.3% vs. five cases in RPD, 6.6%, P > 0.05), which was consistent with other reports (24). Thus, RPD could not significantly reduce the incidence of conversion to open surgery, which may be related to the small number of RPD cases included in our study. The main reasons that 11 cases were converted to open surgery were as follows: the portal vein invasion was too wide for resection and reconstruction under laparoscopy in pancreatic head cancer patients; the second was complicated with severe pancreatitis, and the pancreas was too hard to dissect. These are similar to other studies showing the reasons for conversion to open surgery (22, 28). A retrospective study of European multicenter showed that the risk factors for conversion from MIPD to laparotomy included laparoscopic surgery, large tumour diameter, old age, and pancreas/bile duct tumour. It was also observed that a center with medium flow (10–19 cases/year) has a higher risk of conversion than a center with high flow (more than 20 cases/year) (31). Certainly, there are many factors affecting the conversion; it is not only the patient but also the surgeon’s technique. This question needs further study.

With the assembly of robotic surgical systems, the popularity of laparoscopic equipment, and the widespread use of surgical videos, an increasing number of surgeons and center are carrying out MIPD. Some studies have shown that the learning curve of RPD has advantages over LPD (25, 32, 33). Another meta-analysis indicated that there was no significant difference between robotic and laparoscopic pancreatoduodenectomy in the cases needed to pass the early stage of the learning curve; however, the results of subgroup analysis showed that the RPD learning curve of a single surgeon in a single center was shorter and the early period was easier to conquer (34). A recent network meta-analysis shows that center with high volume can enable patients to obtain better short-term results, that is, fewer complications and shorter postoperative hospital stays (35). This result indicate that those center that are about to perform MIPD or have already performed MIPD but with low flow need to seriously consider whether to continue this operation. From the authors’ experiences, RPD is an easier operation and exhibits less physical consumption. It can significantly shorten the operation time and improve the operation efficiency in the early stage to speed up the recovery of patients. However, after LPD has passed the early stage, the operation time is similar to that of RPD; its unique large range of movement has its own advantages, especially when looking for a suitable section of small intestine during the reconstruction of the digestive tract. In general, laparoscopic and robotic surgery are two similar surgical methods. They have their own advantages in minimally invasive pancreatoduodenectomy. We can choose the appropriate surgical method according to the existing conditions, or the two surgical methods can help each other. We also consider that LPD can help surgeons familiarize themselves with RPD technology faster in our practice, but the specific evidence needs further study. Kim also has a similar view in his article (21). In addition, it needs to be specifically pointed out that the robotic surgery system allows the surgeon to be less tired after the surgery, which has been confirmed by relevant studies (25).

Although minimally invasive pancreatoduodenectomy has been basically developed in high-flow centers, the surgeon’s pursuit of reducing the incision continues. Therefore, some hospitals are also conducting research on single-port minimally invasive pancreatoduodenectomy (SP-MIPD), but the literature is very limited (36–38), and more practice is needed to confirm the necessity and safety of this operation.

This study is a rare single-centers laparoscopic and robotic retrospective study. However, the study also has many shortcomings, such as a small sample size and a single centers. It is inevitable that some random factors will interfere, and there are still many problems to be solved. This study mainly observed the differences between LPD and RPD and may aid in the development of MIPD. We are looking forward to report the long-term results of MIPD in our future studies.

## Data availability statement

The datasets presented in this article are not readily available because the datasets used and analysed during the current study are available from the corresponding author on reasonable request. Requests to access the datasets should be directed to WZ, fcczhaiwl@zzu.edu.cn.

## Ethics statement

The studies involving human participants were reviewed and approved by Ethics Committee of The First Affiliated Hospital of Zhengzhou University. The patients/participants provided their written informed consent to participate in this study. Written informed consent was obtained from the individual(s) for the publication of any potentially identifiable images or data included in this article.

## Author contributions

KZ and KL conceived and designed the article. WZ and KC performed the operation. JY contributed to the data analysis. WL together with KZ contributed to the conception of the study and writing of the manuscript. All authors read and approved the manuscript and agree to be accountable for all aspects of the research in ensuring that the accuracy or integrity of any part of the work are appropriately investigated and resolved. All authors contributed to the article and approved the submitted version.

## Funding

This work was supported by the National Natural Science Foundation of China [grant number 81902336].

## Conflict of interest

The authors declare that the research was conducted in the absence of any commercial or financial relationships that could be construed as a potential conflict of interest.

## Publisher’s note

All claims expressed in this article are solely those of the authors and do not necessarily represent those of their affiliated organizations, or those of the publisher, the editors and the reviewers. Any product that may be evaluated in this article, or claim that may be made by its manufacturer, is not guaranteed or endorsed by the publisher.
